# Reconstruction of the portal vein with 64-slice spiral CT of bile duct obstruction

**DOI:** 10.3892/etm.2013.1138

**Published:** 2013-06-03

**Authors:** YUNBAO XIA, GONGMAO PAN, FENG XUE, CHENGJUN GENG

**Affiliations:** Department of Radiology, People’s Liberation Army 101 Hospital, Wuxi, Jiangsu 214044, P.R. China

**Keywords:** spiral CT, portal venous phase, reconstruction, bile duct obstruction

## Abstract

The aim of this study was to evaluate 64-slice spiral CT image reconstruction of the portal vein in biliary obstruction. A total of 34 clinical patients with biliary obstruction were confirmed by 64-slice spiral CT scanning with portal venous phase multi-planar reconstruction (MPR) of the biliary tract, curved planar reconstruction (CPR), thin-slab minimum-intensity projection (TS-MinIP) and maximum intensity projection (MIP). The reconstructed images were reviewed to further assess the position and cause of the biliary obstruction and to judge the accuracy of diagnosis. The 64-slice spiral CT image reconstruction of the biliary obstructions determined the causes with an accuracy of 88.2% (30/34) and identified the location of the obstruction with an accuracy of 100%. A 64-slice spiral CT reconstructed image of the portal bile duct lesions was generated, which indicated the lesion morphology, location and anatomical relationship with surrounding tissues, secondary biliary stricture and the degree of expansion and is of great clinical value in the characterization and preoperative evaluation of biliary disease.

## Introduction

Bile duct obstruction, usually caused by tumors, calculus, inflammation, trauma or external compression, is a common surgically treated disease. The preoperative localization, qualitative diagnosis and determination of the anatomical relationship between pathological tissue and ambient tissue are important when planning surgery. Therefore, an accurate assessment of the causes and pathological severity of the bile duct obstruction is useful for preoperative surgical planning, reducing the surgical exploration time and for assessing the prognosis of patients. It has been reported that the diagnosis rates of bile duct obstruction by CT, magnetic resonance imaging (MRI) and magnetic resonance cholangiopancreatography (MRCP) are 86.7%, 93.3% and 100%, respectively (1). In the past, the diagnosis rate of bile duct obstruction by common CT was low. Recently, with improvements in scanning speed, and spatial and isotropic resolution, multi-slice spiral CT (MSCT) has been used more frequently in the bile duct system. Due to continuous improvements in the post-processing techniques of MSCT, it is possible to enhance image reconstruction to show the deformation, pathological location, pathological shape and the surrounding tissue lesions clearly using various methods, including rapid thin slice CT scanning, multiple planar reconstruction (MPR) and curved planar reformation. The structure of the common bile duct and ambient tissues is shown more clearly, which further improves the diagnosic accuracy of CT in bile duct obstruction (2–9). In the present study, 34 patients with clinically confirmed biliary obstructive diseases were retrospectively analyzed by reconstructing images of the portal vein from 64-slice spiral CT data. The application value of bile duct reconstruction technology with spiral CT in the diagnosis of obstructive disease is also discussed.

## Materials and methods

### General information

We retrospectively analyzed the spiral CT images of 34 patients with biliary obstructive diseases between May 2009 and February 2010. Twenty cases were male, while 14 patients were female. Aged between 46 and 92 years, the patients had an average age of 67.4 years. Common clinical manifestations included jaundice, abdominal pain, nausea, vomiting, fever and pruritus. The patients in this group were diagnosed by surgical pathology diagnosis or endoscopic retrograde cholangiopancreatography (ERCP). This study was approved by the Ethics Committee of the People’s Liberation Army 101 Hospital (Wuxi, China). Signed informed consent was obtained from each patient prior to examination.

### Equipment and inspection methods

A GE 64-slice spiral CT instrument (GE Medical Systems, Waukesha, WI, USA) was used to scan from the liver top to the L3 level for both plain and multi-phase scanning. A trigger scan was used for the hepatic arterial phase and the trigger point was set at the abdominal aorta. The trigger threshold was set at 120 Hu. The portal venous phase was ∼70 sec and the lag phase was 180 sec. The scan parameters were set as follows: voltage, 120 kV; current, 400 mA; screw pitch, 0.984:1; rotation period of bulb tube, 0.6 sec; and layer thickness, 5 mm. Contrast medium (70–90 ml, Ultravist, 300 mg/ml; Schering Pharmaceutical Co.Ltd., Guangzhou, China) was injected at the speed of 3.5 ml/sec and 1000 ml water was consumed 10–15 min prior to inspection. All original data of the portal venous phase (70 sec) were used for thin-layer reconstruction in the standard mode. The reconstruction thickness was 0.625 mm. The images were sent to a workstation (Advantage workstation, AW4.4; GE Medical Systems) for observation and post-processing analysis. According to the pathological condition, we selected from the following reconstruction methods: MPR, thin-slab minimum intensity projection (TS-MinIP), curved planar reconstruction (CPR) and maximum intensity projection (MIP).

## Results

### Comparison of CT diagnosis and clinical results

A total of 34 patients with biliary obstructive diseases were confirmed by surgical pathological diagnosis or ERCP. The CT diagnosis prior to surgery of 30 of the patients was in accordance with the last clinical result (Table I): 2 cases of hilar cholangiocarcinoma, 6 cases of gallbladder carcinoma invading the common hepatic duct, 8 cases of periampullary adenocarcinoma and carcinoma of the head of the pancreas, 2 cases of common bile duct cancer, 1 case of duodenal papillary adenoma, 2 cases of duodenal papilla diverticula with stenosis of the lower common bile duct and 8 cases of intrahepatic and extrahepatic bile duct calculus. In one case, the pancreaticoduodenal vein surrounded the common bile duct in the shape of semi-circle and compressed it, leading to a slight narrowing of the common bile duct. The CT diagnosis prior to surgery of 4 patients did not agree with the surgical results: CT reconstruction prior to surgery of one patient suggested that ampulla space-occupying caused secondary expansion of the pancreatic and bile ducts, while surgical results showed that it was pancreatitis (Table II). The CT reconstructed image prior to surgery of another case suggested a slight expansion of the pancreatic and bile ducts, but it was later confirmed to be ampullary carcinoma by surgery. CT inspection prior to surgery of 2 cases suggested a slight expansion of the intrahepatic and extrahepatic bile duct, but was verified to be common bile duct negative calculus.

The 64-slice spiral CT bile duct reconstructed images of the 34 patients in this group had an coincidence rate of 88.2% (30/34) for judgment of the obstruction cause and a relevance rate of 100% for judgment of the obstruction location.

## Discussion

**Figure 1.**
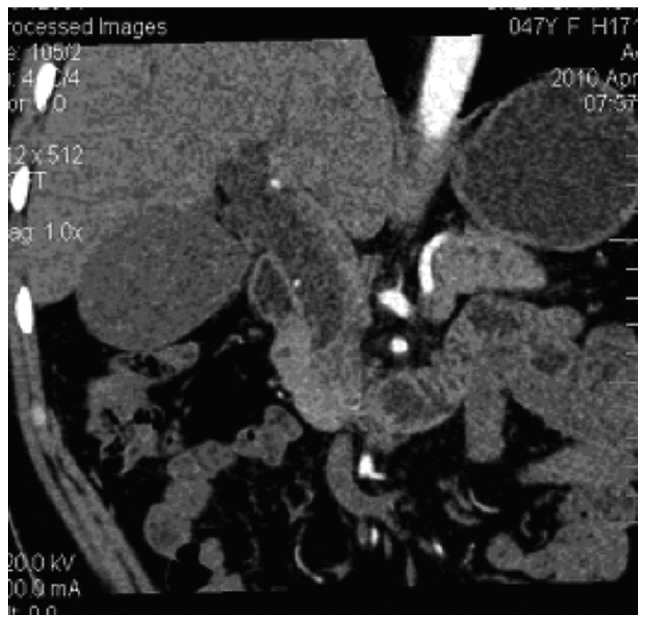
Coronal section construction a patient in the arterial phase.

**Figure 2.**
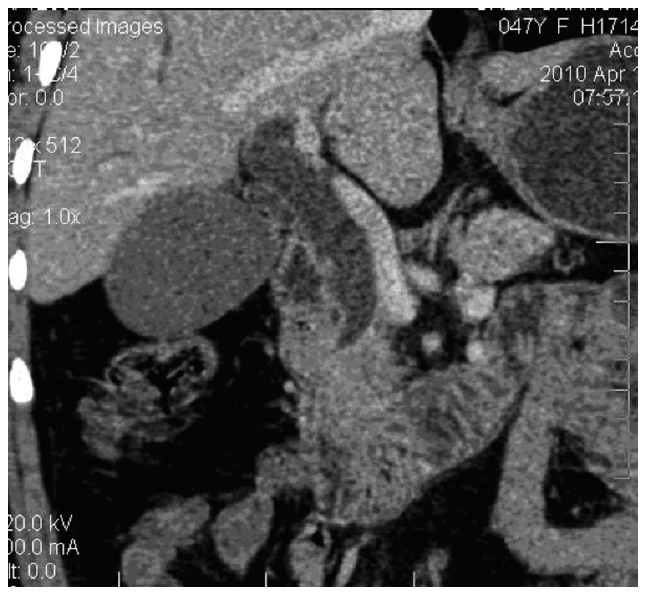
Coronal section construction of the same patient as in Fig. 1 in the venous phase. Comparison of MPR in the arterial phase and portal venous phases showed that the portal venous phase was superior to the arterial phase due to the clear contrast between relatively low-density bile in the bile duct and ambient tissues. MPR, multi-planar reconstruction.

**Figure 3.**
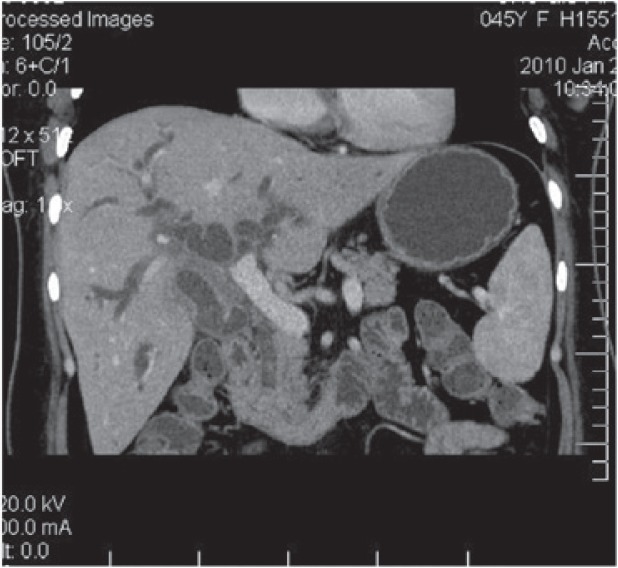
Portal venous phase coronal section MPR showed an abnormal density shadow with an irregular shape of relatively low-density soft tissue in the porta hepatis area, which blocked the common bile duct, invaded the intrahepatic right and left bile ducts and expanded the intrahepatic bile duct in the right and left liver lobes. There was also a relatively high-density calculus at the extremitas anterior of the common bile duct. MPR, multi-planar reconstruction.

**Figure 4.**
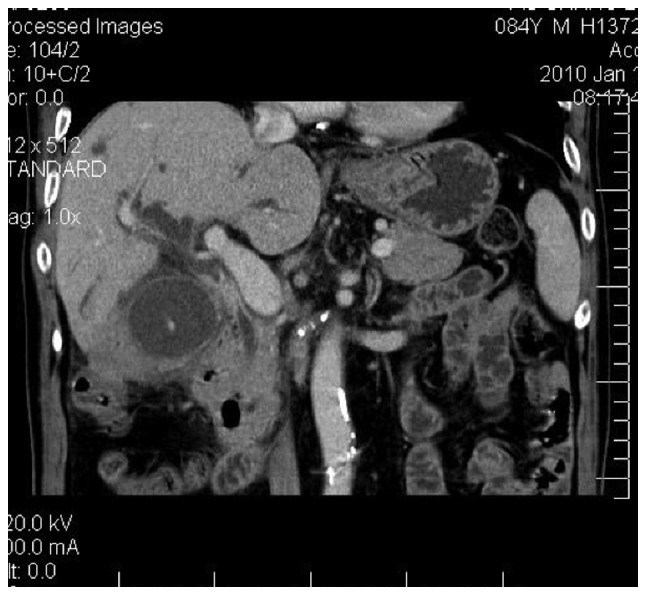
Gallbladder cancer with secondary common bile duct metastasis. The common bile duct wall thickened significantly (shown as relatively homogeneous moderate enhancement), which narrowed the lumen.

**Figure 5.**
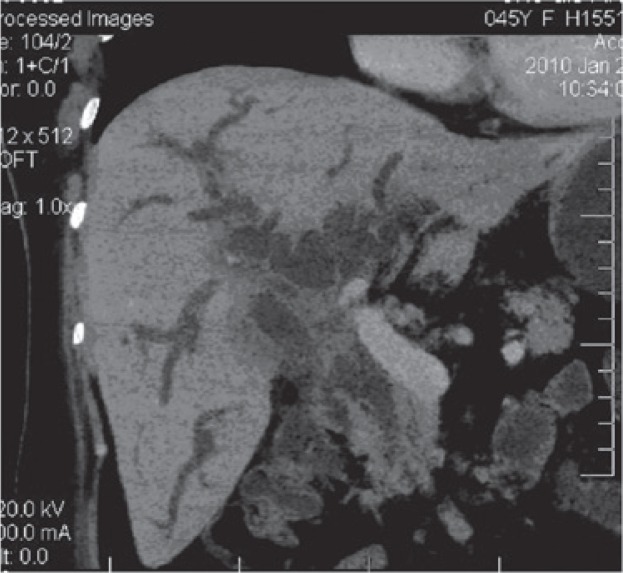
TS-MinIP coronary section showed that the porta hepatis bile cellular cancer invaded the common bile duct and the right and left hepatic ducts, with secondary intrahepatic bile duct expansion. TS-MinIP, thin-slab minimum intensity projection.

**Figure 6.**
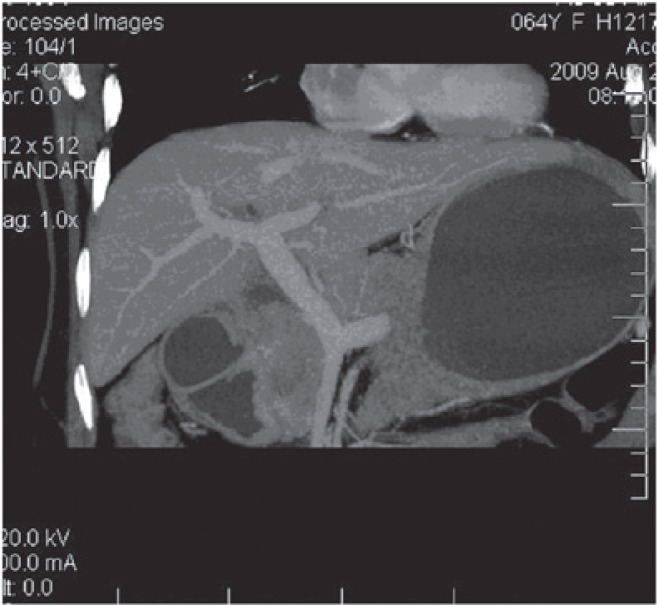
Common bile duct cancer MIP coronary section showed a soft tissue lump in the common bile duct. MIP images showed that the portal vein was normal and was pressed slightly by the common bile duct without invasion. MIP, maximum intensity projection.

**Figure 7.**
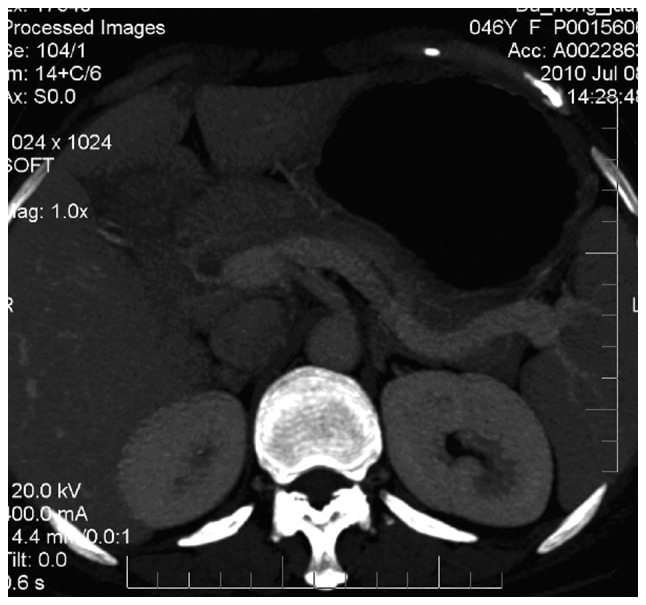
MIP in the portal venous phase. MIP, maximum intensity projection.

**Figure 8.**
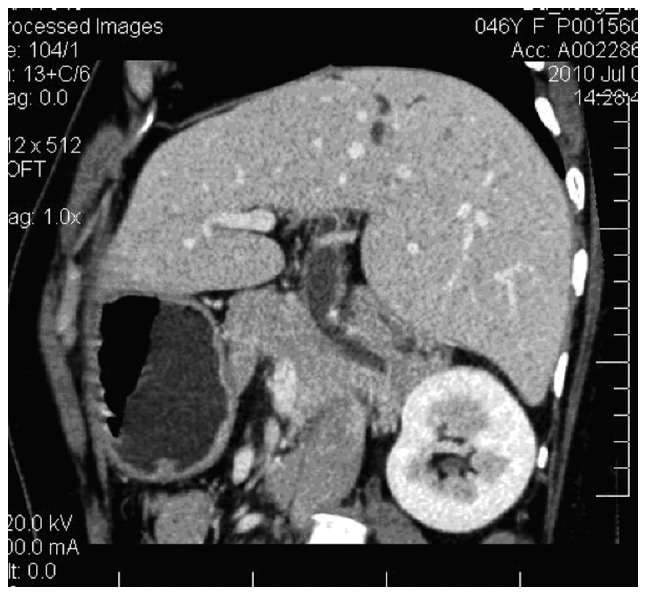
MPR in the portal venous phase of the patient shown in Fig. 7. MIP and MPR, performed respectively in the portal vein phase, directly showed that the pancreaticoduodenal vein semi-surrounded and adjoined the common bile duct, which led to a slight narrowing of the common bile duct and a secondary expansion of the common bile duct and part of the intrahepatic bile duct. MPR, multi-planar reconstruction; MIP, maximum intensity projection.

The 64-slice spiral CT imaging technique, with its high scanning speed, spatial resolution, isotropic resolution and image clarity, showed anatomical relationships clearly. The relationship between the bile duct and the ambient soft tissue structure was displayed well by bile duct reconstruction in the portal vein phase. Due to the significant enhancement of the portal vein near the bile duct and liver parenchyma in the portal vein phase compared with the arterial phase, the contrast between relatively low-density bile in the intrahepatic and extrahepatic bile duct and enhanced ambient soft tissue was clear. The reconstructed images of the bile duct in the portal vein phase were markedly superior to those in the arterial phase (Figs. 1 and 2) (10). Based on the pathological properties, different imaging methods, including MPR, CPR, Ts-MinIP and MIP, were used in the reconstruction. Optimum reconstruction images were chosen and turned to any angle that showed the anatomical configuration and heteromorphosis of the bile and pancreatic ducts clearly and vividly. Contiguous pathological and adjacent tissues were observed for location diagnosis and qualitative diagnosis. Through three-phase enhancement scanning, we were able to confirm the enhanced features of pathological changes, including benignity or malignancy, violation degree, lymph node metastasis, intrahepatic metastasis, vascular embedding and ascites, which may indicate the feasibility of tumor staging and surgical resection (11–22). Through portal vein phase MPR of porta hepatis cholangiocellular carcinoma, we were able to observe that the enhanced portal vein served as a foil to the irregular shape of the porta hepatis soft tissue which blocked the common hepatic duct, invaded left and right hepatic bile ducts and caused the intrahepatic bile ducts of the right and left lobes of the liver to expand (Fig. 3). MPR revealed the bile system with high spatial and isotropic resolution, high imaging speed and no manual cutting. When the bile system is observed, the ambient structure is also observed and any angle is available for assessment. Portal vein phase MPR of gallbladder carcinoma with secondary common hepatic duct metastasis showed significant thickening of the common bile duct wall with homogeneous moderate enhancement, which caused the bile duct lumen to become narrow (Fig. 4). TS-MinIP was superior to traditional cross section images in displaying bile duct expansion and measuring the diameter. As an auxiliary diagnosis, TS-MinIP further confirmed sign of conventional axial images, provided more diagnosic information and improved the confidence of radiologists. Although TS-MinIP does not provide more information than cross-sectional images, it may provide the clinician with a valuable three-dimensional anatomical image of the bile duct. Similar to MRCP, TS-MinIP may determine the location of the obstruction accurately (Fig. 5). TS-MinIP not only showed a high-density calculus and expansive intrahepatic and extrahepatic bile duct, but also displayed a soft tissue density negative calculus in the common bile duct (5). Various routes and rotation angles of CPR may be chosen to display all or the majority of the right and left bile ducts, *ductus cysticus*, common hepatic duct and common bile duct in the same plane. Multiple reconstruction may be performed to comprehensively show the whole morphological structure of the bile duct and clearly show the obstruction location, expansion around the obstruction, the extent of the obstruction and the relationship with ambient tissues. An advantage of the application of CPR is that while traditional CT axial images are not able to show the bile duct continuously, CPR is able to show stenosis of the bile duct more directly and measure the extent of the stenosis (7). As its major advantage, MIP may be used to assess whether the portal vein is invaded by a tumor and the relationship between blood vessels and bile ducts (Fig. 6). In one case, portal vein phase MIP showed that the portal vein was normal and was slightly pressed by a common bile duct carcinoma without invasion. MIP and MPR, performed respectively in the portal vein phase, directly showed that the pancreaticoduodenal vein partially surrounded and adjoined the common bile duct, which led to a slight narrowing of the common bile duct and a secondary expansion of the common bile duct and part of the intrahepatic bile duct (Figs. 7 and 8).

**Figure 9.**
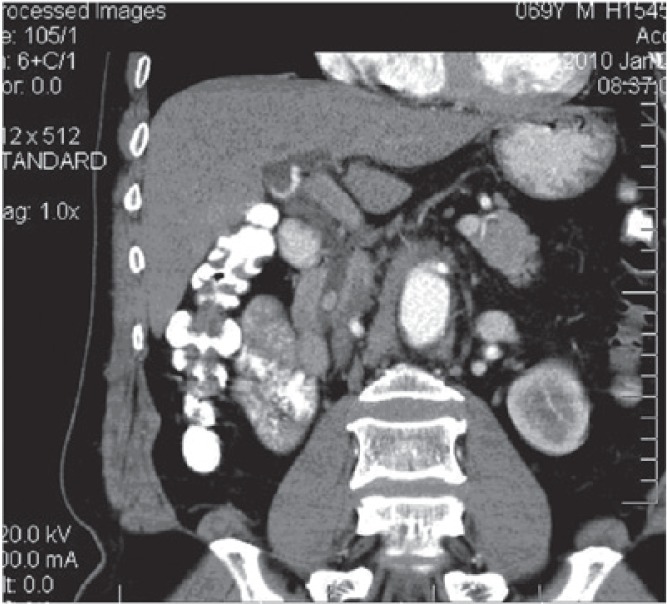
MPR showed a small oval high-density shadow in the common bile duct, which was diagnosed as a common bile duct calculus with secondary expansion of the extrahepatic bile duct. MPR, multi-planar reconstruction.

**Figure 10.**
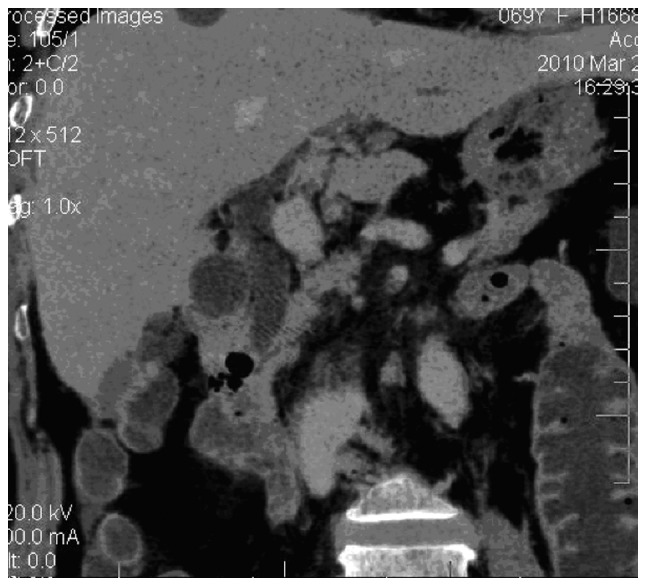
Ts-MinIP showed that the duodenal papillary diverticula presented as a papillary low-density pneumatosis shadow, with inflammatory stenosis of the extremitas anterior of the common bile duct. TS-MinIP, thin-slab minimum intensity projection.

**Figure 11.**
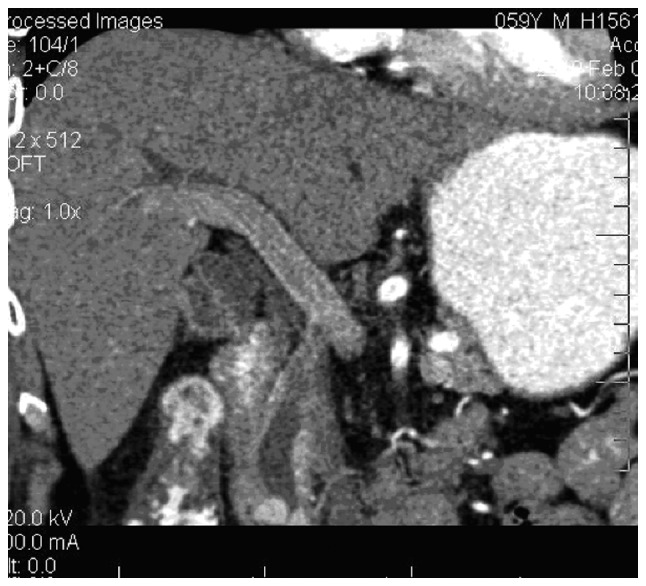
Duodenal papillary cancer oval homogeneous enhanced shadow (diameter 1.2 cm) at the duodenal papilla narrowed the extremitas anterior of the common bile duct.

64-slice spiral CT, with a higher-density resolution, is able to show any density, including that of gas, fat, liquid and calculus, which contributes to a qualitative diagnosis of the obstruction position (Fig. 9). MPR almost completely displays the anatomical relationship between the common bile duct and its ambient tissues and clearly shows the anatomical position of positive calculi in the common bile duct. Bile duct calculi are often accompanied by bile duct inflammation. By enhancing the scanning of the portal vein phase, the enhancement effect of the bile duct wall was shown to be significant. Therefore the calculus with isodensity in the intrahepatic bile duct is able to be identified. For the calculus with isodensity in the intrahepatic bile duct, the CT diagnosis rate was improved due to the sharp contrast to liver parenchyma enhancement after strengthened scanning and the characteristic presence of limited expansion of the intrahepatic bile duct. Bile duct inflammatory stenosis often migrates gradually. The tube wall thickens equally and its edge is relatively smooth, without soft tissue lumps. The portal vein phase is superior to other phases in distinguishing soft tissue lump shadows (Fig. 10). For extrahepatic bile duct cancer or gallbladder carcinoma invading the extrahepatic bile duct, 64-slice spiral CT manifested irregular thickening of the bile duct wall, lumen stenosis obstruction and a soft tissue lump. The thickened tube wall and soft tissue lump presented regular or irregular enhancement. The enhancement extent was more marked in the vein phase than in the arterial phase. The bile duct above the obstruction expanded remarkably. For ampullary cancer, CT showed a lump shadow in the ampullary area and pancreatic head soft tissue. The bile duct was shown to be ‘cut off’ and the bile duct above the obstruction and the pancreatic duct expanded. Based on the tissue source, the enhancement methods of the ampullary cancer focus were varied. For one patient with duodenal papillary adenoma (Fig. 11) in this group, focus enhancement in the arterial phase was clearer than that in the portal vein phase. The efficiency of 64-slice spiral CT reconstruction was not evident for bile duct expansion. When the contrast medium was intravenously injected, reconstruction of MPR, Ts-MinIP, CPR and MIP in the portal vein phase was relatively improved for bile duct three-dimensional images compared with that in other phases, due to the significant enhancement of the portal vein near the bile duct and liver parenchyma and the density contrast between low-density bile in the bile duct and the enhanced bile wall.

Notably the efficiency of 64-slice spiral CT reconstruction was not evident for bile duct expansion. The thickness of the fixed bed will result in much interference. If the fixed bed is thick, it will shade the focus of infection on the side of obstruction and extrahepatic bile duct. If it is thin, the location of pathological changes is not shown fully, particularly in Ts-MinP and MIP images. Image noise levels were high due to the thinness of the layer. The qualitative challenge of missed diagnosis and misdiagnosis of small ampullary foci in this group remains to be solved, which limited the correct diagnosis. The objective reason for this is that the anatomical structure of the ampulla is so complex that relatively small foci are difficult to recognize. The subjective reason is our insufficient experience. The normal slightly inward-protruding anatomical structure of the duodenal papilla of 1 patient in this group was mistaken as a small tumor when we observed images of the coronal view and inclined coronal view. Therefore, ampullary disease imaging should be further explored to improve comprehensive analysis, reduce misdiagnosis rate and improve the precision of early stage diagnosis. The density of negative calculi is similar to that of bile, so CT imaging rarely shows a density contrast between them, which leads to misdiagnosis. Therefore, the negative calculus diagnosis rate of CT is lower than that of MRCP and ERCP.

In conclusion, 64-slice spiral CT reconstruction images in the bile duct portal vein phase are able to clearly show the bile duct structure with obstruction expansion, situation of bile duct wall, ambient anatomical structure and the location of the obstruction. 64-slice spiral CT is capable of affirming the causes of the majority of bile duct obstructions. Therefore, 64-slice spiral CT is worthy of application and promotion in the diagnosis of bile duct obstructive diseases.

## Figures and Tables

**Table I.** Diagnostic accordance of the patients.

Category	Diagnostic accordance
Total number	30
Hilar cholangiocarcinoma	2
Gallbladder carcinoma invading common hepatic duct	6
Periampullary adenocarcinoma and carcinoma of head of pancreas	8
Common bile duct cancer	2
Duodenal nipple department adenoma	1
Intrahepatic and extrahepatic bile duct calculus	8
Duodenal papillary diverticula with stenosis of lower common bile duct	2
Pancreaticoduodenal varicosity compressing common bile duct	1

**Table II.** Diagnostic discordance of the patients.

Category	Diagnostic discord
Total number	4
Pancreatitis	1
Periampullary adenocarcinoma	1
Radioparent calculus of common bile duct negative stone	2
